# The disservice of publishing preliminary results based on a premature hypothesis – Semmelweis’ ordeal revisited

**DOI:** 10.1007/s11019-025-10257-8

**Published:** 2025-02-13

**Authors:** Niels Lynøe, Niklas Juth, Anders Eriksson

**Affiliations:** 1 Centre for Healthcare Ethics, Karolinska Institutet, Stockholm, Sweden; 2 Centre for Research Ethics and Bioethics, Department of Public Health and Caring Sciences, Uppsala University, Uppsala, Sweden; 3 Department of Clinical Sciences, Forensic Medicine, Umeå University, Umeå, Sweden

**Keywords:** Premature results Thomas, Premature hypothesis, Historical controversy, Evidence-based medicine, Theory impregnated observations, Karl Popper, Kuhn

## Abstract

In an interesting article, Dr Zuzana Parusniková claimed: (i) that Semmelweis’ colleagues did not recognise the importance of his animal experiments, (ii) that the resistance to Semmelweis’ hypothesis and results was due mainly to applying mono-causality and (iii) Semmelweis inability to communicate, (iv) that the New Vienna Medical School applied evidence-based medicine, and (v) that the philosophy of Karl Popper is the best interpretation of Semmelweis’ scientific approach. Here, we present some factual aspects of Semmelweis’ text from 1861 and discuss Dr Parusniková’s claims against this backdrop. We conclude that Semmelweis might intentionally have abstained from communicating his hypothesis and results between 1847 and 1849 – including the results from his animal experiments – as he thought that they would eventually be understood and accepted. Semmelweis’ hypothesis was that cadaveric matters and decaying particles were the cause of childbed fever and increased maternal mortality. This hypothesis might have been controversial, but we claim that the major reason for the resistance was eminence-based and induced by the publication of preliminary and suboptimal results, based on a premature version of his hypothesis. If the New Vienna Medical School had been influenced by evidence-based medicine, we believe that Semmelweis’ empirical results would have been accepted - as they were based on an almost randomised controlled trial - and if the results had not been associated with his hypothesis but instead had focused on a black box procedure. We agree that the philosophy of Popper might be appropriate when analysing Semmelweis’ scientific approach when abandoning low-level theories. However, to understand the resistance against Semmelweis’ hypothesis and results, it is not sufficient to refer to a Pickwickian discussion; a Kuhnian framework is more adequate.

## Introduction

Dr Zuzana Parusniková published an interesting and thought-provoking analysis of Semmelweis’ theoretical reasoning and the hostile reception of his suggested use of chlorine handwash to decrease maternal mortality in childbed fever (CBF) (Parusniková 2023). As she states, historians and philosophy of science researchers have made many different interpretations of what Semmelweis did, the intentions and reasoning behind his choices and the development of his hypotheses, and the reception of the empirical results suggested to support his theory. Some have focused on the applied empirical methods (Hempel 1965, Lipton 2004), others on causality and mechanisms or on the negative reactions after the publication of preliminary results in late 1847 (Gillies 2005; Semmelweis in Codell Carter 1983).

We agree with Dr Parusniková that certain aspects of Semmelweis’ reasoning when developing his hypothesis could be understood within the framework of Karl Popper’s philosophy, but we find several claims that deserve comments. Dr Parusniková stated that:

i. Semmelweis’ colleagues did not recognise the importance of his animal experiments conducted in 1849–1850.
ii. The resistance against Semmelweis’ results was due mainly to the idea of mono-causality.
iii. The resistance was also related to Semmelweis’ inability to communicate with his colleagues.
iv. The New Vienna Medical School (see below) was an example of evidence-based medicine (EBM).
v. The most plausible way to understand Semmelweis’ research, methods and reasoning is through the philosophy of Karl Popper.

In the following, we discuss these five points. First, we present how we understand the factual aspects of Semmelweis’s scientific approach, his hypotheses and his experimental results.

## Relevant background and facts about Semmelweis

### Maternal mortality and autopsies

Before the introduction of autopsies, the maternal mortality rate in Vienna was steady at approximately 1% in a sample of 65,000 deliveries. During this period (1789–1822), anatomy and pathology were taught using leather models (Kadar et al. 2018). After 1822, the leather models were replaced by dissections of human cadavers in accordance with a governmental decree (Kadar et al. 2018), followed by the introduction by professor von Rokitansky of pathological anatomy for training of physicians and medical students. Already in 1823, the maternal mortality rate had risen to over 7%.

### Division into two clinics, later with differing staff

In 1830, the maternal clinic was split into two, here referred to as the First Clinic and the Second Clinic, which were both initially served by doctors and midwives. The maternal mortality rate remained high and similar in the two clinics (Parusniková 2023; Semmelweis in Codell Carter 1983). In 1834, the autopsy routines were expanded, and in 1836 every deceased patient underwent an autopsy. The purpose of the autopsies was to compare pathological findings with clinical diagnoses. By 1837, the maternal mortality rate had risen to 9.1%. In 1841, the maternal clinic was further divided, so that physicians and medical students (all male) worked at the First Clinic and midwives and midwife students (all female) at the Second Clinic.

As the maternal mortality rate increased substantially at the First Clinic after 1841, Semmelweis began considering possible causes. The monthly mortality rate varied – in 1841 it was 0.74% at its lowest and 22.55% at its highest. Semmelweis tested several hypotheses, including that the cause of CBF was offended modesty, fear or shame due to the presence of males at delivery. Also, cosmic telluric phenomena were considered to have a potential influence, although Semmelweis reasoned that this should have had the same impact also upon the Second Clinic (Semmelweis in Codell Carter 1983).

### The development of Semmelweis’ hypothesis

Semmelweis had noted that women who delivered ‘in the street’ – often assisted by a midwife – were less often affected by CBF than those at the hospital. He also noted that women who delivered prematurely “*became ill much less frequently than ordinary patients*” (Semmelweis in Codell Carter 1983). Further, Semmelweis observed that women at the First Clinic whose dilatation period exceeded 24 h fell ill already during or shortly after delivery, whereas *“[a]n equally extended period of dilatation in the Second Clinic did not prove dangerous*”.

Not until his colleague, the pathologist Professor Jacob Kolletschka, died after being cut in a finger during an autopsy did Semmelweis develop the first version of his final hypothesis. Semmelweis recognised an analogy between the autopsy findings in maternal CBF cases and those in Kolletschka’s dead body. Semmelweis concluded that the autopsy findings in CBF were not specific to women after labour, and presented the hypothesis that the cause of CBF was “*cadaveric matters*”.

Semmelweis received permission to conduct a preventive intervention trial by requiring the physicians and medical students to wash their hands in chlorine before entering the First Clinic. This trial was initiated in June 1847.

Results from the last seven months of 1847 were published in late 1847 (Codell Carter 1983). However, three of these seven months (September–November) showed an increased mortality rate which later could be related to e.g., a woman who had a CBF-infected uterine carcinoma. The average maternal mortality rate during these seven months was 3.04%. The rate fell to 1.27% in 1848, but those significantly better results were not published until 1861 (Table 1) (Semmelweis in Codell Carter 1983).

**Table 1:** A somewhat anachronistic analysis (regarding p-values) of five studies conducted in 1753–1872. Apart from the Jenner experiment, the examples were probably inspired by the ‘La Méthode Numérique’ and positivistic philosophy in terms of focusing on empirical observations and the quantification and analysis of observations. The p-values were calculated by us, using Fisher’s exact test

Researcher (year, disease, participants & outcome)	Hypothesis	Design	Results	Hypothesis supported	Reception and interpretation of results
James Lind, 1753, scurvy, *N* = 12, survival	Citrus fruit prevents and cures scurvy	Comparing citrus fruit and five other treatments	Only citrus was curative (*p* =.015)	Yes	Accepted 40 years later; eminence-based resistance (Captain Cook)
Edward Jenner, 1796, smallpox, *N* = 1, survival	Inoculation of cowpox prevents smallpox	Single experiment	Prevention was effective	Hypothesis plausible	Eminence-based acceptance (Napoleon)
Pierre Louis, 1836, pneumonia, *N* = 77, mortality rate	Late bloodletting has better outcome than early bloodletting	Experiment comparing times	Non-significant (*p* =.098)	No	Late bloodletting slightly better or already cured?
Semmelweis, 1847 (last 7 months), CBF mortality, C1 = 1,841 vs. C2 = 3,306, CBF mortality rate	Chlorine handwash prevents cadaveric matters from causing CBF	RCT, comparing C1 with C2	C1: 3.04% (2.36–3.82) *versus* C2: 0.97% (0.64–1.3)	No, hypothesis immature and hence suboptimal results	Eminence-based resistance to the hypothesis, (normal variation) but neutral to chlorine wash
Semmelweis, 1848 (all year), CBF, *N* = 3,556 vs. *N* = 3,219, CBF mortality rate	Chlorine handwash prevents cadaveric matters + decaying particles from causing CBF	RCT, comparing C1 and C2	C1: 1.27% (0.91–1.63) *versus* C2: 1.33% (0.64–1.72)	Yes	Apart from “Koran of the puerperal theology“ almost no reaction in 1848
Joseph Lister, 1870, postoperative sepsis, *N* = 75, sepsis mortality rate	Antiseptic procedure focused on the air over the surgical wound decreases mortality rate	Results were compared with historical controls	Intervention significantly better than control means (*p* =.005)	Yes	Rapidly accepted, surgeons’ reputation improved

P-values < 0.05 are considered significant. P-values < 0.0001 are presented as proportions with 95% confidence intervals within brackets. C1 = the First Clinic, C2 = the second clinic, CBF = childbirth fever, RCT = randomised controlled trial

### An important auxiliary hypothesis

Almost all women in labour were clinically examined shortly after examination of the woman with the CBF-infected uterine carcinoma, and these women developed CBF and died. Semmelweis then understood that the clinicians and students should also chlorine-wash their hands *between* each individual examination *within* the First Clinic. The cadaveric matter hypothesis was thus broadened to include also “*decaying particles*” from living patients (Kadar 2019; Gillies 2005).

### Publishing premature results was probably a disservice

The CBF-infected cancer patient caused an average maternal mortality rate of 3.04% (95% confidence interval (CI): 2.26–3.82) in the last seven months of 1847 when the results were reported in an editorial by von Hebra who supported Semmelweis - in the “Zeitschrift der Gesellschaft der Ärzte zu Wien” (Codell Carter 1983), and “*[h]eads of maternity clinics in Europe were invited to confirm or refute the results*.” With few exceptions, the responses were negative (Kadar et al. 2018). A typical and comprehensive response came from Professor Levy in Copenhagen, who concluded that: “*His [Semmelweis’] views appear too unclear*,* his observations too volatile*,* and his experience too uncertain to deduce scientific results therefrom*.” (Gotfredsen 1948). Levy’s two main arguments were: (i) that a minor amount of contagious matter from the physicians’ nails was not enough to kill a patient, and (ii) that the empirical data was a result of “statistical fluctuation” (Gillies 2005).

Professor Klein, Semmelweis’ superior, agreed with Levy’s last argument. Klein concluded that the results from the last seven months of 1847 *with* chlorine handwash (3.04%) and those from the first five months (5.6%), when no chlorine was used were due to natural variations. “*Statistical fluctuation*” and “*natural variation*” represented normal scientific reactions when the data were in conflict with the dominating paradigm (Gillies 2005). The suboptimal effect of the chlorine handwash during the last seven months of 1847 could, however, be explained as an effect of the CBF-infected cancer patient. Semmelweis had at that time not yet included “*decaying particles from living patients*” in his hypothesis.

### An unfortunate miscalculation of data

As the CBF mortality rate had already decreased to an average of 5.6% (95% CI: 4.5–6.7), this further corroborated Klein’s scepticism. However, this rate of 5.6% was probably incorrect and should instead have been 7.8% (95% CI: 6.5–9.2). According to Semmelweis’ Table XXIII in his original text in German, presenting the rates of the first five months of 1847, there were 912 births in February, in which 6 mothers died (Semmelweis 1861). According to the table, these figures corresponded to a mortality rate of 1.98%, but this would have been correct for 312 births, not for 912. As the other four months (January, March, April and May) each had ~ 300 births per month, we suggest that the correct number of births in February should be 312. This would mean there were 120 deaths out of 1,534 births during January through May, and a mortality rate of 7.8% (95% CI: 6.48–9.2). Consequently, the mortality rate of 3.04% (95% CI: 2.26–3.82) *with* chlorine handwash during the last seven months of 1847 should be compared with 7.82% (not 5.6%) *without* handwash. This miscalculation was noticed also by Gillies (2005).

### Professor Klein and the new vienna medical school

Professor Klein was described as “dull, unimaginative, but a politically well-connected man who owed his position to bureaucratic patronage” and belonged to the old and conservative guard, not the New Vienna Medical School (Kadar 2019). Several other professors were associated with the New Vienna Medical School, for example Josef Skoda and Ferdinand Ritter von Hebra. Carl von Rokitansky, from whom Semmelweis learned a lot about pathology and autopsy, has been considered “*the Linné of pathological anatomy*” (Codell Carter 1983; Otis-Hidalgo 2020). Such knowledge was essential for the development of Semmelweis’ hypothesis, as it enabled him to recognise that the autopsy findings in the women and newborns were identical to those in Kolletschka, and to conclude that CBF was merely a variety of pyaemia (sepsis). Previously, it was considered a unique disease that only females contracted (Codell Carter 1983). But as also Kolletschka died with similar autopsy findings as in CBF, Semmelweis concluded that even males could be attracted by CBF. Semmelweis’ procedure to systematically exclude possible endemic causes (e.g., fear, shame, self-infection, rough male students, etc.) could be understood in accordance with Karl Popper where conjectures are refuted logically and/or by means of empirical studies. The latter was the case when Semmelweis tested the assumed roughness of medical students when examining the women, or the hypothesis that the priest who gave the dying women the last sacrament scared or terrified women when passing the rooms on the First Clinic (and ringing a bell) – whereas he did not pass the Second Clinic. Semmelweis asked the priest not to ring the bell and not to pass the rooms in the First Clinic, but this had no impact upon the mortality rate on the First nor the Second Clinic (Gillies 2005).

Even though Semmelweis had several local (e.g., Skoda, von Hebra, and von Rokitansky) and also international supporters (e.g., Professor Michaelis from Kiel), the large majority of colleagues were sceptical. This scepticism occurred despite the philosophy of the new Vienna Medical School which was also influenced by the interest in, and focus on, empirical research and observation and classical positivistic thinking by, e.g., Francis Bacon and Auguste Comte (Johansson et al., 2008; Edmonds 2020). There were influences also from Paris by, among others, Pierre Louis, who also tried to assess older medical treatments as, e.g., bloodletting (Table 1) (Morabia 2006). Some members of the New Vienna School actually supported Semmelweis, but proponents of the dominating (sub-)paradigm had difficulties accepting Semmelweis’ hypothesis - if they had been true supporters of EBM they would probably have accepted his results.

### Semmelweis’ successors

Semmelweis was replaced by Dr Carl Braun and later his brother Dr Gustav Braun, both of whom were antagonists of Semmelweis’ theoretical reasoning. The maternal mortality rate slowly increased in the beginning of the 1850’s, peaking in 1854 at 9.1% (95% CI: 8.25–9.95) in the First Clinic (Semmelweis 1861). This increase took place despite the continued use of chlorine handwash – at least in principle, although Semmelweis assumed that the Brauns had influenced the medical students, and perhaps even the physicians, to ignore the instructions on chlorine handwash (Semmelweis in Codell Carter 1983).

Despite a large majority of clinicians and professors in Europe did not accept Semmelweis’ theoretical reasoning and his hypothesis, many adopted his chlorine handwash procedure and the empirical results. In fact, it was already applied in British islands due to the early acceptance of the contagion theory (Gillies 2005). Even the surgeon professor Joseph Lister is claimed to have been inspired by Semmelweis’ chlorine handwash procedure, although Lister himself later denied this (Codell Carter 1983). Lister, however, applied an antiseptic procedure, but instead of focusing on the surgeons’ hands, Lister focused on the air above the wound – quite in accordance with the established miasma theory and hence considered non-controversial also by proponents of the still dominant Galenic paradigm (Johansson et al., 2008).

### A quasi-randomised study design

There were large study samples from both the First Clinic and the Second Clinic. Further, the women were prospectively and randomly allocated to the two clinics through an every-other-day procedure, which could be considered a quasi-randomisation procedure (Kadar 2019). However, from Friday at 4 pm to Sunday at 4 pm, *all* women were allocated to the First Clinic, resulting in 4 × 24 h allocation to the First Clinic and 3 × 24 h to the Second Clinic (Semmelweis in Codell Carter 1983). An almost random and equal allocation to the two clinics regarding relevant aspects such as age, primipara/multipara, delivery complications, duration of labour and delivery, use of forceps, premature delivery, etc., is thus to be expected. This means that the two groups of maternal patients would have been comparable, with a low risk of selection bias (Altman 1991). If the rationale of randomization had been known when the study was conducted, it would have been difficult to claim that the results were due to statistical fluctuations or normal variations. But modern statistics was not introduced until the early 1900’s.

### Semmelweis’ animal experiments

Klein’s argument that Semmelweis’ results represented nothing but normal variations was probably the reason why Semmelweis initiated animal experiments in the spring and summer 1849 and continued them in the spring and summer 1850 (Kadar 2020). The first animal experiments included 9 adult female rabbits that were vaginally inseminated with CBF matter *after* delivery. Autopsy findings for 7 of the 9 rabbits were similar to those for the women who died from CBF. This study was unfortunately discontinued (due to social problems with a coworker), even though the results indicated what Semmelweis wanted to show: What he assumed was neutralised by the chlorine handwash could cause CBF also in an animal. The results of the study the following summer, which was conducted by Professor Brücke, were somewhat ambiguous and he suggested that Semmelweis’ hypothesis could be corroborated or falsified only by continuing the clinical trial (Kadar 2020).

### The exclusion of Professor Klein from an investigating commission

In 1848, one member of the New Vienna Medical School, Professor Josef Skoda, proposed the faculty to nominate a commission to evaluate Semmelweis’ study and include data from those who had delivered “in the street”, where the CBF mortality rate was lower than at the First Clinic. These data were no longer available to Semmelweis after 1848 and he was denied access when requesting them (Codel Carter 1983). The proposal was adopted, but Professor Klein protested, and might have felt offended because he was not elected as a member of the commission. As a consequence, a higher authority (the Ministry) intervened, and the commission was unable to begin its activities. These circumstances might have informed Klein’s decision to deny Semmelweis’ request to extend his appointment (Kadar 2020; Codel Carter 1983).

### What happened when Semmelweis left Vienna?

Semmelweis left Vienna in October 1850 and returned to Budapest, where he in 1855 was appointed Professor of Theoretical and Practical Midwifery at the University of Pest (Kadar 2020). When he began his deeper analysis of the results from 1847 to 1848, he included comparisons with other maternal clinics in Europe. After he left Vienna, the First Clinic only half-heartedly continued the chlorine handwash procedure. As already mentioned, maternal mortality reached a peak of 9.1% in 1854 (Semmelweis in Codell Carter 1983). A significant decrease of CBF mortality after a preventive intervention can indicate causality, and a significantly increased mortality rate after its interruption further increases the probability of causality (Hill 1965). This could be added to the fact that when both doctors and midwives worked at both clinics there was no difference in mortality rate, but when doctors were allocated only to the First Clinic and midwives only to the Second Clinic, the difference became highly significant (Parusniková 2023).

### Semmelweis and his Magnum Opus

In 1861, Semmelweis declared in the preface to his Magnum Opus (“Aetiology, Concept, and Prophylaxis of Childbed Fever’ and ‘Reaction to My Teaching”) (Codell Carter 1983) that he by nature was averse to all polemics, and that he left numerous attacks unanswered. He believed that he could “leave it to time to break a path for the truth” (Semmelweis in Codell Carter 1983). However, after 13 years of silence, this expectation had not been fulfilled. This was the reason why he published his Magnum Opus. Chapter 6 – the final chapter in the English version of this book – is entitled “Reaction to My Teachings: Correspondence and Published Opinions” (Semmelweis in Codell Carter 1983). In 1857–1860, Semmelweis also published seven papers on CBF, but after the publication of his Magnum Opus, he published nothing more on CBF. He published five more papers on other matters in 1862–1865, before his death on August 13, 1865 (Codell Carter 1983).

## General comments on Dr Parusniková’s claims

First of all, one might assume that the Semmelweis’ clinical study, might have been somewhat influenced by the classic positivism expressed by Francis Bacon (1561–1626) and Auguste Comte (1798–1857) (Johansson et al. 2008, Edmonds 2020). Bacon warned against different *idols* (or illusions), which were assumed to influence scientists’ observations. Nowadays, we would instead refer to different types of bias such as cognitive bias, confirmation bias, optimism bias, selection bias, etc. According to Bacon, one could get rid of or neutralise the effect of the idols and make theory-independent observations. To scientists, the most relevant idol was the “Theatre”, which referred to “old philosophical dogma” that influenced scientists’ observations and interpretations of results (Johansson et al. 2008).

Moreover, in the early 1800’s, ”La Méthode Numérique” was developed in Paris in the spirit of enlightenment and spread to Vienna and other European capitals. It thus became common to collect data from different hospitals, e.g., the number of admissions, how many were cured or died, what kind of treatment had been applied, etc. (Johansson et al. 2008). However, the question is whether these new scientific approaches were generally accepted and acknowledged by clinicians and professors outside the enlightened Paris and Vienna. Even today, EBM is not always accepted, if the technology assessed is controversial (Strouse 2016; Lynøe and Eriksson 2020; Squier 2019). Medical societies often belong to what Parusniková (2023) calls “the conservative medical mainstream” – a mainstream which rules until a new generation of scientists takes a different path and adopts new theories (Azoulay et al. 2019). As Gillies (2005) suggested, medicine is not a typical “natural” science. Apart from traditional Kuhnian criteria, a medical paradigm is associated with social consequences of how to prevent and cure diseases as well as boards and authorities which regulate healthcare providers’ duties and values. Apparently, it might be difficult to apply a Kuhnian analysis of medical research and scientific development, but nevertheless some Kuhnian aspects are relevant and might contribute to a better understanding of the Semmelsweis’ case (Gillies 2005). Moreover, following the mainstream consensus is often associated with eminence-based dominance or resistance rather than evidence-based knowledge (Bhandari et al. 2004). In this respect, there is no big difference between 1850 and 2024 (Squier 2019; Lynøe et al. 2019).

### Comments on Parusniková’s five claims

(i) The claim that Semmelweis’ colleagues did not recognise the importance of his animal experiments.

Skoda, one of the proponents of Semmelweis, argued that if Semmelweis succeeded to conduct animal experiments which indicated causality, continued clinical studies would not be necessary. Also Semmelweis believed that if cadaveric matters and decaying particles from living could be shown to cause CBF in animals, he could convince Klein that his hypothesis was plausible (Parusniková 2023; Semmelweis in Codel Carter1983). However, the first animal study was questioned because the experiment was interrupted (Kadar 2020), and the second study showed ambiguous results. Hence, Semmelweis was recommended by Professor Brücke to instead continue the clinical trial (Kadar 2020).

Dr Parusniková claimed that Semmelweis’s colleagues did not recognise the importance of his animal experiments, but this does not seem to be correct. Some might have relied on Professor Brücke’s (eminence-based) interpretation of the results (Bhandari et al. 2006) and thought that animal experiments were not the right way to proceed. For example, it was unknown how and when to insert cadaveric matters into the vagina of an animal. Semmelweis inserted cadaveric matter *after* the rabbit’s delivery, but in humans it was inserted via the physicians’ hands *before* or *during* delivery, according to Semmelweis’ hypothesis (Kadar 2020). Our conjecture is that part of the explanation why Semmelweis’ animal experiments were not taken more seriously was the misconception that medicine is an exact science (Martin 2020), or that many did not understand when mechanisms and causality could be established (Hill 1965; Koch 1878).

Therefore, the two deviating rabbit cases were attributed to a flaw in Semmelweis’ experimental methodology, rather than shifting the responsibility on the current (miasma and contagion) theories. Popper was against Kuhn’s development in “a normal scientific phase” and would probably have claimed that the scientific development during 1847–1850 was in an extraordinary or preparadigmatic phase. But according to Kuhn, results that are in conflict with the current dominating paradigm during a normal scientific phase indicates that it is the researcher who is to be blamed and made responsible; in an extraordinary phase on the other hand, it is the theory that is held insufficient or at least must be questioned (Kuhn 1981). As indicated, Popper preferred focusing merely on the extraordinary scientific phase with its permanent revolutionary development (Popper 1981). But as claimed, permanent revolution or mini-revolution has nothing to do with the Kuhnian scientific revolution; the former is rather about “another day at the office” from a Kuhnian perspective (Goodwin 2015). We will develop this further below.

Even though Skoda encouraged Semmelweis to conduct animal experiments in order to clarify causality, Semmelweis could have communicated his results better, e.g., by publishing the first experiments and results. There were, however, also other plausible reasons why his animal experiments were questioned, as indicated by, among others, Professor Brücke.

(ii) The claim that the main reason of the hostile resistance towards the results was due to mono-causality.

In her introduction, Parusniková states that despite several issues and critics, Semmelweis conjectured the germ theory before the “existence of germs was established by laboratory experiments (Lister, Koch) some twenty years later” (Parusnikovà 2023).

This is, however, not quite correct as various microorganisms were described already in 1676 by the Dutch lens grinder Antonie van Leeuwenhoek (Porter 1999). But even though this was an important discovery, microorganisms were considered as a *product* of the disease process and not a *cause* (Johansson et al., 2008). Accordingly, it took approximately 200 years before Louis Pasteur launched his hypothesis that microorganisms could actually *cause* infection diseases. Inspired by Pasteur – who was a chemist and hence not influenced by the contemporary medical paradigm and its resistance against mono-causality - Robert Koch developed his four criteria for identifying that a specified microorganism was the cause of a certain disease by means of his anthrax experiments (Koch 1878).

Moreover, in contrast with the reception of Semmelweis’ chlorine handwash preventive (low-tech) procedure, Lister conducted a study in which he compared the mortality rate before and after the introduction of an antiseptic measure. Lister succeeded in showing that the mortality rate decreased after the introduction of an antiseptic fluid atomizer machine (high-tech), suggested to clean the *air* above the surgical wound. Focusing on the air above the surgical wound was in accordance with the miasma theory, and might have directed attention from the physicians’ hands to the air, making it difficult to blame the surgeon. Since the surgeons’ reputation was rather low after the introduction of anesthesia in 1846 and a subsequent increase in surgical procedures and of the mortality rate, Lister’s preventive procedure contributed to both a decrease of the mortality and of an increase of the surgeons’ reputation. Contrary to the reception of Semmelweis’ results, this was - together with the fact that Lister’s results were published 15 years later and after the presentation of Pasteur’s hypothesis – a plausible reason why Lister’s antiseptic procedure was accepted rather promptly.

Dr Parusniková might be right when she suggested that the resistance against Semmelweis’ results was mainly due to his claim of mono-causality, i.e., that one condition only explained an effect. Mono-causality was not yet accepted even though Henle had introduced two theories in 1840 - the miasma theory and the contagion theory - or both in collaboration. The contagion theory was, however, highly controversial on the European continent, where the miasma theory dominated. The contagion theory could be considered a forerunner of the microbiological framework and Semmelweis’ cadaveric matters, and perhaps also made the results difficult for proponents of the miasma theory to digest.

It has also been claimed that the reason why Semmelweis failed to convince his peers was not the fact that he promoted an alternative hypothesis about disease causality; Semmelweis failed because he promoted a mash-up of various contemporary theories associated with the two main theories – the miasma theory and the contagion theory (Henle 1840, Wootton 2007). As also illustrated by Parusniková, CBF and several other diseases were associated with psychological factors such as stress, fear, offence to modesty, as well as supernatural factors. Psychosomatic causes were seriously discussed as aetiology during the 1880’s and many years forward, exemplified by the French author, Marcel Proust who suffered from hay fever and asthma, and got the diagnosis “*neurasthenia*”. The underlying cause of his symptoms was assumed to be that the 6 year old Proust appealed to receive more attention from his mother (Lindgren 2024). The diagnosis of neurasthenia was often applied when there was no identifiable biological explanation – as peptic ulcer was considered to be a psychosomatic disease before Helicobacter pylori was accepted as a cause (Johansson et al. 2008).

However, there were also other reasons why Semmelweis’ hypothesis and results caused such a hostile reaction, particularly in late 1847 when the preliminary results of Semmelweis’ trial were published.

Parusniková claims that the introduction of chlorine handwash applied in 1847 resulted in a “*dramatic drop*” in CBF mortality rate this year (Parusniková 2023). It was, actually not a dramatic drop due to the miscalculation of the mortality rate and due to the patient with a CBF infected tumour. Compared to the results in the end of 1848 one cannot claim that the results from 1847 imply a “dramatic drop”. As indicated the issue was rather that these results were partly based on miscalculations and on an immature hypothesis, not on the broadened hypothesis applied from 1848, when chlorine handwash was performed also *between* patients *within* the First Clinic. If Semmelweis had not miscalculated the mortality rate in February 1847, and if von Hebra had waited with his publication until the end of 1848, the mortality rate would have truly represented a “dramatic drop”. But when comparing the reported mortality rate of the first 5 months with the last 7 months, the results from 1847 might very well have been understood as normal variations (Gillies 2005).

Dr Parusniková quoted Semmelweis as having claimed that decaying matter is *“…the universal etiological factor for all cases of CBF without a single exception*”. Parusniková writes that “*this insistence on mono-causality was hard to accept not only for the conservative medical mainstream but also according to the New School of evidence-based medicine in the Vienna hospital…”.* Mono-causality might have been offensive as it was not in accordance with the old Galenic dogma nor Henle’s presentation focusing on the miasma and contagion theories (Henle 1840).

There was also another issue – associated with the Galenic philosophy – related to the scientific status of medicine. According to Galenos, medicine was a “*natural science*”, initially devoid of religious influences or other supernatural phenomena. Medicine became united by the humoral pathology (Fåhraeus 1944), with an assumed interaction between the microcosmos (human beings) and the macro-cosmos (planetarian positions and constellations). As an example, the occurrence of syphilis was thought to be traceable to an exact date (25 November 1484), when there was a conjuncture of Saturn and Jupiter in the sign of Scorpio and the House of Mars (Fleck 1979). Medicine was viewed as an “exact science”, like mathematics, which meant that inter-observation variations and biological variations were not yet considered or accepted (McCormack et al. 2020).

The assumption that medicine and other empirically based sciences were “exact sciences” might during the 1800’s still have influenced medical scientists like Professor Klein and the mainstream of conservative physicians (Martin 2020), as well as some of those belonging to the New Vienna Medical School (Codell Carter 1983). Obstetricians at that time were aware that women in labour could die from conditions other than CBF, e.g., prolonged delivery, primipara delivery, multiple birth and prematurity. However, mortality in such conditions was estimated to constitute less than 1% of the total maternal mortality, as this was the mortality rate before the introduction of routine autopsies (Semmelweis in Codell Carter1983).

Another important issue was the fact that if Klein and Semmelweis’ colleagues had accepted the results of the preventive intervention trial, this would have attributed these physicians with responsibility for the death of thousands of women and newborn babies. Responsibility, risk of decreased reputation, conflict of interests and power plays were probably important aspects of Klein’s – and others’ - resistance to Semmelweis’ results. When comparing the mortality rate at the First Clinic with that of the Second Clinic, it would also have been highly embarrassing to admit that midwives were better and safer than the physicians, even delivery “in the streets” appeared to be much safer (Codell Carter 1983). All these aspects might have influenced Klein’s decision not to renew Semmelweis’ appointment and thus getting rid of not only Semmelweis but also all associated issues.

Accordingly, we agree that mono-causality might partly have contributed to the resistance against Semmelweis’ results, but his hypothesis (not necessarily the results), the responsibility issue, the publication of the miscalculations, and the premature hypothesis in 1847, probably contributed even more to this resistance. Moreover, Lister’s high-tech anti-septic machine focusing on the air and hence in accordance with the miasma theory, also disguised the responsibility issue (Johansson et al. 2008). Semmelweis’ preventive intervention, on the contrary, was not compatible with the miasma theory and focused on the physicians’ hands and thus on their responsibility. Accordingly, we argue that except for the mono-causality issue, the social and legal consequences probably also contributed to the resistance against the Semmelweis’s hypothesis and results.

(iii) The claim that the resistance was related also to Semmelweis’ inability to communicate with his colleagues.

According to Gillies (2005), Semmelweis in his Magnum Opus promoted himself and put down some of those who criticized his work. This book was also described as too long and too self-promoting. If the Magnum Opus had been shorter, more colleagues might have read it (Gillies 2005), which indicates that Semmelweis had suboptimal pedagogical skills when arguing with others. However, regarding the claim that Semmelweis was *unable* to communicate with his colleagues, it seems documented that his silence after late 1849 was largely intentional and related to Klein’s hostile reaction to Semmelweis’ results from 1847 (Semmelweis in Codell Carter 1983).

In the preface of his Magnum Opus, Semmelweis stated that he believed that time would make it unnecessary to respond to the criticism of the 1847 results (Semmelweis in Codel Carter 1983). This was something that the physicist Max Planck also stated when he realiseld that relevant and rational arguments had no effect on antagonists of the quantum theory. According to Planck, mankind would have to wait until the antagonists had passed away and a new generation had become accustomed to the new theory.

Semmelweis was silent for 13 years, but when time did not solve the controversy, he lost patience and decided to respond to the criticism. This response was presented in a separate chapter in his Magnum Opus (Semmelweis in Codell Carter 1983). Had Semmelweis waited silently for another 13 years, time would have solved the controversy, facilitated by the introduction of the microbiological paradigm (Johansson et al. 2008).

In the chapter entitled “*Reactions to My Teachings*”, Semmelweis mentioned letters from both proponents and opponents, as well as other publications which referred to the chlorine handwash intervention. In total, he had had at least twenty discussions with directors and professors from Amsterdam, Berlin, Copenhagen, Edinburgh, Graz, Kiel, London, Munich, Prague and Vienna (Semmelweis in Codell Carter 1983). The longest discussion was with Semmelweis’ successor, Dr Carl Braun, who was an open opponent of Semmelweis’ theoretical reasoning and his hypothesis. Even though Semmelweis’ chlorine handwash was supposed to remain in use after he left his position, he suspected that Braun’s negative attitude towards the chlorine handwash procedure influenced the attitudes of medical students (and probably also his colleagues), so that they neglected the procedure (Semmelweis in Codell Carter 1983).

Accordingly, we agree with Parusniková that Semmelweis’ communication with his colleagues in Europe was probably suboptimal and insufficient after the publication of his results from the last seven months of 1847 compared with the miscalculated number of births during the first five months. However, we do not find that he was *unable* to communicate with his colleagues as illustrated in his Magnum Opus. Moreover, his silence could likely have been a result of his hope that time would solve the problems. Semmelweis might also have understood that von Hebra’s publication of premature results had been a disservice as it was also based on a hypothesis not yet including the important auxiliary hypothesis about decaying particles from living. Although this auxiliary hypothesis was a precondition for the significantly more promising outcome during 1848, Parusniková did not mention this important difference between the 1847 results and the 1848 results (Parusniková 2023). Semmelweis did however not publish the 1848 results until the Magnum Opus in 1861. He then began communicating to both critics and supporters of the CBF theory as well as the results of his clinical studies. So, when Semmelweis realized that time would not solve the scepticism against his empirical results, he responded and communicated with many colleagues (Semmelweis in Codell Carter 1983). Focusing on the bantering comments, describing his results as e.g., “the Koran of the puerperal theology” indicated that many physicians might have interpreted his writing as metaphysical speculations (Codell Carter 1983). The scepticism probably did not disappear until the microbiological paradigm was established and a normal scientific phase was developed, demonstrating how microorganisms were identified as a cause of various diseases. The normal scientific phase, however, also disclosed its limitations by interpreting what we currently recognize as (vitamin) deficiency diseases as caused by microorganisms (Johansson et al. 2008).

Again, we agree that Semmelweis’ communication was suboptimal, but disagree that he was *unable* to communicate with them. For example, he had a largely rational discussion with, among others, Professor Levy from Copenhagen – even though they disagreed about Semmelweis’ hypothesis (Godtfredsen 1948, Codell Carter 1983).

(iv) The claim that the New Vienna Medical School was an example of evidence-based medicine (EBM).

Dr Parusniková suggested that the New Vienna Medical School was an expression of EBM. There can be different views about this, depending on what one believes should be included in EBM and its aims. As proposed by Dr Parusniková, it seems reasonable to claim that Semmelweis’ clinical trial was quasi-randomised. Accordingly, the Semmelweis trial might be considered to be a large randomised controlled trial (RCT) with a low risk of selection bias (Altman 1991; Chalmers 2011).

But, is it really reasonable to refer to EBM in relation to the methods of these early empirical researchers and before modern statistical procedures were introduced? Representatives of the Cochrane Collaboration in Oxford considered that the English navy physician James Lind (1716–1794) was the first medical researcher who conducted an evidence-based trial (Table 1) (Clarke et al. 2018). In this sense Parusniková might be right.

What was traditionally recommended (by Captain Cook) to cure scurvy was malt and sauerkraut, as well as other treatments like bloodletting, putting a piece of turf into the patient’s mouth to inhibit the bad quality of sea air (in accordance with the miasma theory), and providing citrus fruits. Lind selected 12 sailors who suffered from scurvy and were in a very bad state, and divided them into 6 pairs receiving different treatments, with one group receiving citrus fruits. After one week of citrus fruit supplement, both these two sailors had recovered completely, and could take care of the remaining 10. However, it took more than 40 years before the British Navy started to provide citrus fruits to prevent or treat scurvy. It has been claimed that Lind did not have sufficient authority – at least not within the Navy and not compared with Cook (Clarke et al. 2018). This might again illustrate the difference between EBM on the one hand, and eminence-based medicine and resistance on the other (Bhandari et al. 2004).

It is relevant to note that deliberate randomisation procedures and statistical methods were not introduced until the early 1900’s – and, in medicine, a full century after Semmelweis’ study. The first deliberately conducted RCT within medicine was performed by Austin Bradford Hill in 1948 (Hill 1965). Therefore, it is somewhat anachronistic to claim that EBM was established in Vienna already in the 1840’s. Surely, Parusniková was aware of this anachronism and rather wanted to claim something more fundamental: that Semmelweis’ research was also an example of a scientific approach which was *in accordance with modern* EBM – as the term had not been coined at the time.

One factor influencing this in the 1850’s was that few physicians were likely to understand statistical reasoning and the risks of various types of bias. Minimising the risk of bias is crucial when conducting evidence-based studies, regardless of whether they are assessing a new treatment, diagnostic accuracy or a preventive intervention (Schünemann et al. 2020).

The manner in which Semmelweis arrived at his hypothesis/theory could very well be described as a Skoda-inspired method, or what would currently be considered a Popperian framework. Semmelweis *abandoned* quite a few hypotheses about the cause of CBF. He applied both logical reasoning (*modus tollens*) and supported this with empirical data presented in the often-ignored tables in his Magnum Opus. This was actually the case when Semmelweis considered a common (Galenic) hypothesis that the increased CBF mortality rate at the First Clinic was due to atmospheric influence. If the increased mortality rate on the First Clinic was due to atmospheric influence, the mortality rate would have been the same at the Second Clinic. Another example was that the high mortality rate at the First Clinic was due to crowdedness. Also in this case, Semmelweis applied both logical reasoning and empirical data - as it was actually more crowded in the Second Clinic - crowdedness as a cause could be abandoned. One hypothesis was abandoned solely by empirical examinations before and after the number of medical students had been halved; it was namely the hypothesis that male medical students (and especially foreign students) were rougher than midwives when examining the women in labour. After this reduction of male students, the CBF mortality decreased immediately, but three months later no difference was longer discernible. Although the empirical results did not really falsify the theory, it was simply abandoned because it was less probable (Semmelweis in Codell Carter 1983).

Accordingly, and in analogy with the scurvy case, we agree with Parusniková that Semmelweis’ methods could be described as a forerunner of EBM. If the New Vienna Medical School were influenced by EBM, their representatives would at least have been impressed by Semmelweis empirical results from 1848. But such representatives would probably not have considered whether the results supported Semmelweis’ hypothesis and theoretical considerations; they would rather have interpreted the empirical results as moderate or low risk of bias indicating moderate or high scientific evidence that chlorine handwash could prevent CBF mortality.

(iv) The claim that the most plausible way to understand Semmelweis’ research, methods and reasoning is through the philosophy of Karl Popper.

Dr Parusniková claimed that best way to understand Semmelweis’ research and methods is based on the philosophy of Karl Popper. As indicated above this seems reasonable, as Semmelweis tested several low-level theories more or less derived from the Galenic dogma and abandoned them in order to develop a new, more relevant and plausible theory.

Moreover, when he identified the analogy between the autopsy findings in the CBF women and the autopsy findings in his colleague Kolletschka, Semmelweis suggested as a hypothesis that “cadaveric matter” was the causative agent. This reasoning was also supported by his autopsy observations in deceased newborns whom he supposed had been infected when passing through the birth canal during a protracted delivery (Semmelweis in Codel Carter 1983), and where prematurely newborn babies (smaller and hence passing the birth canal more easily and quick) were less influenced (Gillies 2005). In September through November 1847, he observed that “decaying particles” could be spread also *between* patients *within* the First Clinic. Both physicians and students were therefore required to chlorine-wash their hands *between* examinations of individual patients. The hypothesis was thus broadened to include both “cadaveric matters” and “decaying particles from living”. When the results of his first trial were questioned by Klein and others in late 1847, Semmelweis wanted to obtain empirical support from the broadened hypothesis by applying the preventive handwash procedure during 1848 and actually succeeded with his aim (Table 1). But Klein was still not convinced and hence Semmelweis performed his animal experiments. Unfortunately, these results were also questioned and he was recommended to continue his clinical trial. However, this became impossible as Klein did not renew Semmelweis’ appointment. The actions of Klein probably reflect the real world of conflicts of interests, power plays, preferences, responsibilities, reputation of the First Clinic, etc.

As several authors have indicated, medicine is not quite comparable with natural science associated with exact sciences, because there are social and legal consequences of how to prevent and cure diseases. A medical paradigm also takes into account that there are institutions and regulative authorities which can influence the dominating paradigm and the development of scientific knowledge, and also influence the healthcare providers (Gillies 2005; Goodwin 2015). These considerations are probably in accordance with how Thomas Kuhn would have described a scientific environment. Kuhn analysed and learned from the history of scientific development within the natural sciences, that such development sometimes undergoes more dogmatic phases – also referred to as a normal scientific phase in which scientists are expected to follow the scientific mainstream (Kuhn 1979). According to Popper, Kuhn’s “normal scientific phase” is dogmatic and should be avoided because it does not allow critical thinking and the attempt to falsify dominating theories, and instead allows proponents to defend accepted dogmas within the current paradigm (Popper 1981). Popper suggested that scientists should approve critical thinking and a kind of permanent revolutionary inclination by trying to falsify the current theories adopted by the actual paradigm. In this sense, Semmelweis possibly acted in the spirit of Popper.

Poppers’ resistance towards verification is comprehensible when he – for example – refers to psychoanalysis where denying something very strongly can be interpreted as if the actual claim is true (Popper in Lakatos and Musgrave 1981). As suggested when discussing Parusnikovà’s former claim (iv), Popper had a problem regarding verification and falsification. In exact sciences such as e.g., theoretical physics (applying thought experiments), astronomy, logic and mathematics it might be possible to verify and falsify a certain claim but in observation-based knowledge much can be questioned. As argued above, medicine is not an exact science (Martin 2020), and even though Semmelweis might have used *modus tollens* to abandon some old hypotheses, he based his reasoning mainly on observation-based knowledge or empirical observation based on more or less auxiliary hypotheses. Hence, it is not possible to verify nor to falsify a theory, even by randomised controlled trials (Johansson et al. 2008).

When Semmelweis was appointed at the Vienna maternity clinic in 1846 he might have found himself in a responsibility crisis when he discovered the high mortality rate at the First Clinic as compared with that of the Second Clinic. This difference created a problem for him, and he found that the dominating contemporary scientific paradigm was unable to explain the difference. Semmelweis thus aimed to develop a new hypothesis which could explain the high mortality rate, and in the longer term aid to prevent morbidity and mortality. This enables us to describe Semmelweis as being in an extraordinary scientific phase, between an old paradigm partly associated with Galenic dogmas and new scientific ideas and strategies, where scientific anarchy could mean that “*anything goes*” (Feyerabend 1993; Gillies 2005). All researchers mentioned in Table 1 might be seen as pioneers in this manner.

Rather promptly, however, Semmelweis found himself in a trench warfare between the defenders of an old paradigm – represented by, among others, Professor Klein - and himself and his supporters, trying to test a preventive measure and a new theory; a theory which was incomprehensible according to the current normal scientific framework or paradigm. If Semmelweis instead had tried to apply a *black box procedure* focusing on *input* (chlorine handwash) and *outcome* (decreased CBF mortality) and abstained from any mechanism theory, he might have succeeded. Black box procedures can be considered as a pragmatic solution to paradigmatic resistance associated with controversial theories (Johansson et al., 2008). Maybe such a pragmatic procedure is a better strategy to manage resistance associated with controversial theories, rather than avoiding the Kuhnian concept of “normal scientce”.

The distinction between a “normal science” and extraordinary science is important, as scientific progress and environment differ greatly. A progressive example of a normal scientific phase would be at hand when Robert Koch had introduced his method and criteria to identify a microorganism as the cause of a certain disease (Koch 1878), and one researcher after the other identified various microorganisms as the cause of various specific diseases (Johansson et al., 2008). Between 1878 and 1906, around 20 pathogenic microorganisms were identified as pathogens of specific diseases. This normal scientific phase had, however, its limitations when scientists who adopted the microbiological paradigm tried to solve vitamin deficiency diseases by searching for microorganisms (Johansson et al. 2008). Although one might claim that science under Klein’s regime was on the verge of an extraordinary phase, the conservative and eminence-based resistance is best understood within a Kuhnian framework vis-a-vis the normal phase of scientific development (Bhandari et al. 2004; Kuhn 1981).

Popper took it for granted that there is no problem of communication between persons accepting different theories. Kuhn on the other hand claimed that communication is only partial, that there is no neutral observation language, and that comparison of different theories is a problem of translation (Johansson 1975). Moreover, Popper stated: “*I do admit that at any moment we are prisoners caught in the framework of our theories; our expectations; our past experiences*,* our language. But we are prisoners in a Pickwickian sense: if we try*,* we can break out of our framework at any time*.”

The benevolent Pickwickian view might be an idealistic interpretation of how science could - or should - function. The Semmelweis case and other examples from the real world illustrate that scientific discussions both within the same (sub-)paradigm and between different (sub-) paradigms can be much more difficult. Metaphorically, it can be likened to scientific warfare, a warfare where rational arguments are replaced by threats, intimidations, bullying, accusations of scientific misconduct and negative *ad hominem* arguments. Ultimately, a scientist may be shunned and marginalised from the scientific society by its members (Strouse 2016; Squier 2019; Lynøe and Eriksson 2020; Leventhal et al. 2023; UK Supreme court). This bears no striking resemblance to a Pickwickian discussion club and shows that there is a difference between an idealistic school of thought like positivism and more realistic descriptions of how scientific (sub-)paradigms sometimes function. In the Semmelweis case and many others, the descriptions by Kuhn seem more plausible and the Popperian vision of how scientific work should be done appears too idealistic.

## Concluding remarks

In our view, the main issue regarding the reception of Semmelweis’ result seems to have been the premature publication of preliminary results in an editorial comment and that an immature hypothesis gained ground among some of his supporters. Inviting responses and reactions to the preliminary results was probably the reason why Semmelweis’ reasoning and results were questioned, and why the reaction became so negative. The 1847 hypothesis encompassed only “cadaveric matter”, but this did not explain the problems with a CBF infected uterine carcinoma, causing an increased mortality rate despite chlorine handwash. In 1848, however, the inclusion of “decaying particles from the living” solved these problems. The results of the 1848 trial based on the broadened hypothesis and widened indication of chlorine handwash would probably have been more appreciated (and statistically more convincing, *p* =.000018) than those of the 1847 trial.

We agree that Semmelweis’ scientific approach could to some extent be understood within the framework of Karl Popper, but the scientist who actually influenced Semmelweis was probably Skoda. As regards scientific controversies, Popper might be characterised as somewhat too idealistic, at least when suggesting to resolve scientific controversies in a Pickwickian manner.

A scientific discussion can and should of course be performed as a Pickwickian Club meeting, but this is possible only if there are no major scientific controversies or conflicts within the research group. The Pickwickian Club metaphor is not always applicable when trying to understand a scientific controversy (Freudenthal 2000; Lynøe et al. 2020) with its complexity of miscommunication, conflict of interests, personal preferences, power plays, bullying and shunning a scientist if not following the mainstream – or worse, stabbing a dissenter in the back in order to get rid of him/her, like in the Semmelweis case. Such controversies are ongoing within several medical areas, and we do find that the philosophy of Kuhn represent a more realistic description of what unfortunately occurs in some scientific environments.

However, we suggest that rather than eradicating the Kuhnian normal scientific phase, researchers could at times avoid premature promotion of new controversial theories and to instead apply a pragmatic black box procedure, focusing on the evidence of, e.g., an RCT. Or, to follow the strategy (intential or not) of Joseph Lister who disguised his real theory behind the dominating miasma theory. Such a strategy might in the first place neutralise resistance towards the new theory.
